# 3D Printing in Digital Prosthetic Dentistry: An Overview of Recent Developments in Additive Manufacturing

**DOI:** 10.3390/jcm10092010

**Published:** 2021-05-07

**Authors:** Josef Schweiger, Daniel Edelhoff, Jan-Frederik Güth

**Affiliations:** 1 Department of Prosthetic Dentistry, University Hospital, Ludwig-Maximilians University Munich, 80336 Munich, Germany; Daniel.Edelhoff@med.uni-muenchen.de; 2 Poliklinik für Zahnärztliche Prothetik, Center for Dentistry and Oral Medicine (Carolinum), Goethe-University, 60596 Frankfurt am Main, Germany

**Keywords:** 3D printing, digital one-piece casting, multi-material 3D printing, graphic 3D models, 3D printing using composite resin, digital pressing technology, 3D printing using zirconia, hybrid production

## Abstract

Popular media now often present 3D printing as a widely employed technology for the production of dental prostheses. This article aims to show, based on factual information, to what extent 3D printing can be used in dental laboratories and dental practices at present. It attempts to present a rational evaluation of todays´ applications of 3D printing technology in the context of dental restorations. In addition, the article discusses future perspectives and examines the ongoing viability of traditional dental laboratory services and manufacturing processes. It also shows which expertise is needed for the digital additive manufacturing of dental restorations.

## 1. Introduction

The pace of development in digital dental manufacturing has become impressive. High levels of productivity and accuracy of fit have been achieved by subtractive processes, while additive processes (3D printing) are increasingly coming to the fore. Combinations of different manufacturing methods—such as laser sintering plus CNC machining or digital design and 3D printing plus analog ceramic pressing—display the enormous potential [1,2].

## 2. Current State of Technology

### 2.1. A Rationale for Digital Manufacturing and 3D Printing in Dentistry

Fundamental changes in society are also affecting dental technology, like any other area. One of these changes is the shortage of skilled workers; the number of trainees in dental technology is continuously decreasing [3] even as the demand for dental prostheses remains high due to changing demographics [4,5]. In addition, patients are increasingly subject to time constraints created by rising expectations in the workplace, limiting their ability to undergo dental procedures. The digital transformation can help us meet these challenges, as digital processes are often characterized by their efficiency. Digital processes in the dental laboratory provide for greater accuracy and reproducibility (precision) as well as improved material properties and user comfort.

The interesting combination of a digital working environment and an analog craft makes dental technology attractive to young people looking for a varied and diverse work experience. Many dental laboratories are already managing the balancing act between craftsmanship and the digital world, tradition and disruption, and existing values and necessary changes. 3D printing as a digital manufacturing process is an important aspect of this development. In simplified terms, the process can be described as follows: The dental technician creates a digital data set on the computer (computer-aided design, CAD) and then designs a three-dimensional object whose data are transferred to the 3D printer, where it is converted into a physical object.

A major advantage of all additive processes is that three-dimensional objects can be designed and realized on screen to allow for an almost unlimited variety of shapes and levels of complexity. One aspect that has received little attention is that the mechanical and esthetic properties of the object to be printed can still be influenced during the 3D building process. This is not possible with subtractive manufacturing, where the material properties are defined by the manufacturer of the prefabricated blank. This customization option and the fact that digitally designed objects are available more quickly and easily, or even at lower cost, makes additive manufacturing a cornerstone of digital dentistry (Dentistry 4.0) [6,7,8,9,10,11].

### 2.2. History of 3D Printing

The first industrial-level units for additive manufacturing (commonly termed 3D printing) appeared on the market in the early 1980s. Pioneers of 3D printing include Charles W. Hull (founder of 3D Systems), S. Scott Crump (founder of Stratasys), and Hans J. Langer and Hans Steinbichler (founders of EOS). The first 3D printer was patented by Charles W. Hull in 1986 [12]. At the time, 3D printers were mainly used for rapid prototyping.

However, the technology advanced rapidly in the ensuing years. Following the expiration of the patent for the fused deposition modeling (FDM) process [13] in 2009, the 3D printers began to make enormous inroads into the consumer sector. This dynamic was ultimately carried over to the dental sector. Printing units became smaller and cheaper, and their fields of application changed. The range of printable materials expanded to include plastics, metal, ceramics, and even human tissue. Rapid-prototyping processes can be categorized by the type of materials used (plastics, metals, or powder).

### 2.3. Nomenclature and Classification of Additive CAD/CAM-Based Manufacturing

In additive manufacturing (AM) processes, objects are produced layer by layer on the basis of three-dimensional models. The term used in common parlance as a synonym for all additive processes is *3D printing* [14].

According to the EN ISO/ASTM 52,900 terminology standard, an AM process is the “process of joining materials to make objects from 3D model data, usually layer by layer, as opposed to subtractive manufacturing methods” [15].

**Figure 1:**
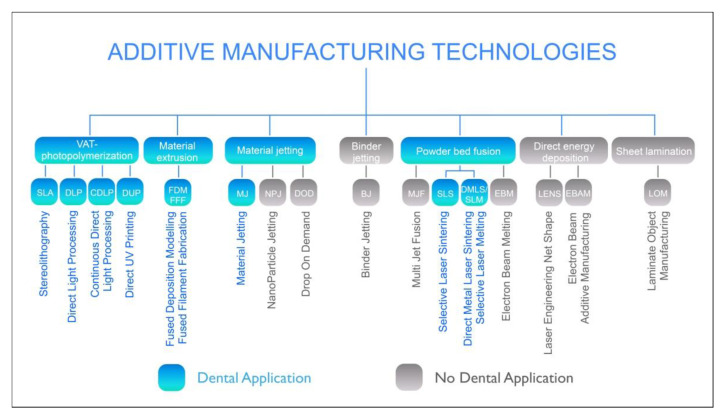
Overview of the existing process categories in additive manufacturing. (According to EN ISO 17296-2).

EN ISO 17296-2 describes the process fundamentals of additive manufacturing. It also provides an overview of the existing process categories, although such an overview can never be comprehensive, given the dynamic development of innovative technologies (Figure 1).

The following seven process categories can be distinguished within additive manufacturing [16]:

- Vat photopolymerization (**VPP**)
- Material extrusion (**MEX**)
- Material jetting (**MJT**)
- Binder jetting (**BJT**)
- Powder-bed fusion (**PBF**)
- Directed energy deposition (**DED**)
- Sheet lamination (**SHL**)

## 3. The Status Quo of Dental 3D Printing

Additive manufacturing has raised high expectations. Its market potential is thought to be considerable. The Gartner Hype Cycle [17], providing a powerful snapshot of current trends, reviews public attention to a specific technology (such as 3D printing) in the context of its development over time. The Hype Cycle is divided into five parts. For the innovation trigger, a potential technology breakthrough and media interest trigger significant publicity as commercial viability is unproven. At the peak of inflated expectations, the topic is hot, and unrealistic expectations are fueled by excessive enthusiasm. This is followed by the trough of disillusionment and the slope of enlightenment, in which public interest has decreased, but the technology is being improved. At the plateau of productivity, the technology is recognized and maturing.

**Figure 2:**
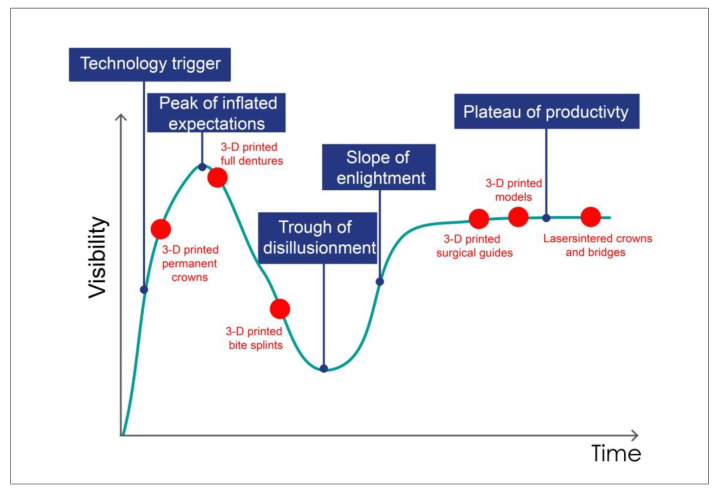
Dental 3D printing follows the characteristics of the Gartner hype cycle.

Dental 3D printing follows this hype cycle (Figure 2). The Gartner analysis predicted in 2014 that 3D printing would take about 10 to 15 years to full adoption. This could be roughly true for the dental sector, if probably not as much as marketing claims would suggest. Neutral institutions should be tasked with attenuating inflated forecasts and supporting the continuous establishment of the technology as a function of the state of research and development, and to modulate expectations. Yet, the potential is, in fact, immense. Dental technicians and dentists should familiarize themselves with 3D printing technology and objectively assess possible areas of application.

### Common Processes in Dental 3D Printing

The technology is not entirely new. Additive manufacturing has been established in the dental sector for almost 20 years, represented, for example, by the laser sintering (selective laser melting, SLM) processes of Bego Medical (Bremen, Germany) and EOS (Krailing, Germany). When presented for the first time in November 2002, this technology for printing metals caused a sensation. Experts recognized the enormous potential of this technology. Moreover, SLM enjoys worldwide acceptance as the basis for manufacturing metallic structures (such as crowns, bridges, or clasp-retained cast-metal frameworks). Stereolithography (SL), too, has been used in the dental industry for many years, for example, in the production of surgical templates (drilling guides). Stereolithography is based on point-by-point solidification within a resin vat (epoxy resins, acrylates) by means of a laser beam or with the aid of blue-light LEDs (digital light processing, DLP). Until a few years ago, 3D printers for dental applications were the preserve of industry or large manufacturing centers, given the considerable capital outlays required, but for some time now, many printers have come down to within reach of “regular” dental laboratories. Moreover, industry outsiders are entering the dental market and offering additive manufacturing technologies. Using comparatively inexpensive equipment, dental laboratories can now realize objects made of acrylics or composite resins to be used in the preparatory stages of a workflow, such as jaw models or surgical templates.

## 4. Dental Indications and Applications of 3D Printing

Not all additive technologies are suitable for use in the dental laboratory or practice. The following sections will discuss indications and applications for 3D printing that are sensible and economical to use in dental technology or else have great future potential. These will be differentiated not on the basis of technologies but on the basis of the materials used, i.e., metals, plastics, and ceramics.

### 4.1. Additive Manufacturing and Metals

Additive manufacturing using metal alloys has been successfully used in the dental sector since 2002. The use of laser sintering in the dental field represented a revolution in the processing of non-precious alloys at the time [18].

#### 4.1.1. Laser Sintering of Crowns and Bridges Made from Non-Precious Alloys

**Figure 3:**
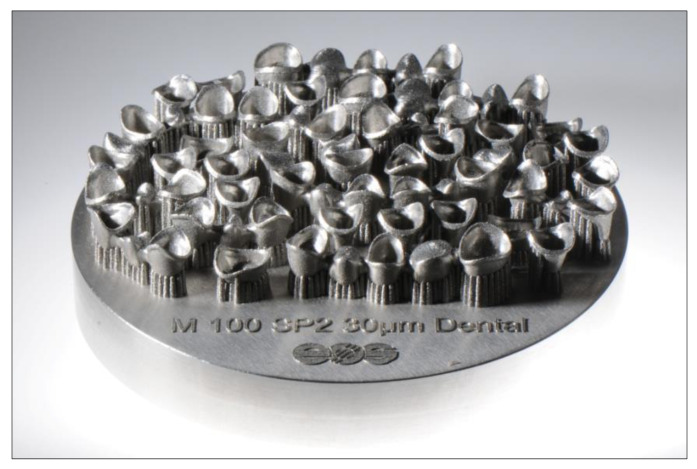
Lasersintered CoCr Crowns and Bridges.

Laser sintering has now become a standard process for the production of CoCr crowns and bridges [19]. By optimizing post-processing after the actual building process, it is now possible to manufacture absolutely stress-free and accurately fitting non-precious alloy frameworks even for larger bridge spans. The large number of units that can be positioned on a single platform has reduced the production time per unit to a few minutes (Figure 3). The procedure is extremely cost-effective and is well established when it comes to fixed restorations made of non-precious alloys.

To generate stress-free restorative frameworks, the build platforms are summarily subjected to a thermal post-treatment in a downstream processing step before the individual restorations are separated from the build platform. Most production centers automate this step. The support structures are then removed by manual finishing.

The physical and mechanical properties of laser-sintered non-precious alloy crown and bridge frameworks are comparable to cast restorations [20,21]. The rougher surface compared to cast or milled restorations actually has a positive effect on the cementation of laser-sintered crowns and bridges. Inside the crown and at the crown margins, laser-sintered restorations exhibit small but macroscopically visible ledges parallel to the *z*-axis of the building process. Nevertheless, the fit of laser-sintered crowns is within the clinically acceptable range [22]. Other studies have found that laser-sintered CoCr-alloy crowns have even a better marginal fit than casted CoCr-alloy crowns [23,24,25]. Ceramic veneers are very easily applied to laser-sintered frameworks as their rougher surface makes them highly wettable by the opaquer.

#### 4.1.2. Laser Sintering of Clasp-Retained Cast-Metal Frameworks

Clasps are one of the oldest forms of denture retention [26]. Clasp-retained dentures, also referred to as one-piece cast dentures, are a simple form of restoration and allow a wide range of variations, making them universally applicable [27]. For more than 100 years, clasps have been a proven means of retaining removable dentures in the presence of withdrawing forces—for example, when speaking or chewing—and of distributing occlusal forces as evenly as possible to the residual teeth and soft tissue. In 1930, Dr. F. E. Roach wrote in the Journal of the American Dental Association [28]: “The clasp is the oldest and still is and probably will continue to be the most practical and popular means of anchoring partial dentures.”

**Figure 4:**
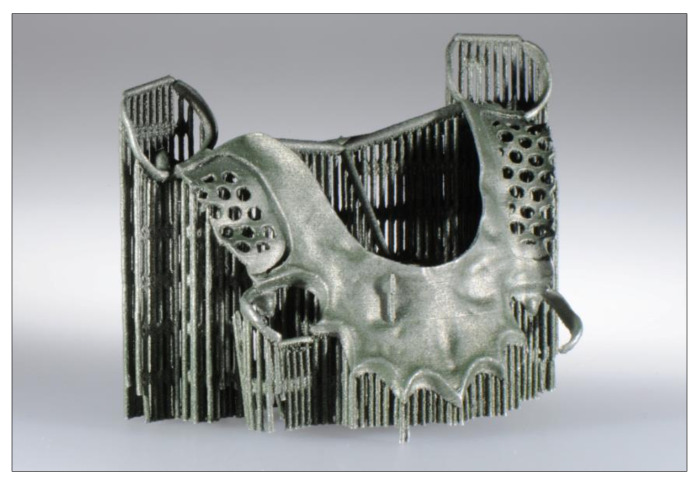
Laser sintered removable partial denture with support structures.

The introduction of digital techniques for the production of dentures, such as computer-aided design/computer-aided manufacturing (CAD/CAM) and additive manufacturing techniques, allows one-piece prostheses to be planned digitally and manufactured subtractively using CNC milling units, or additively, using 3D printing [29]. Here, we can distinguish between indirect and direct fabrication methods. In the indirect method, the frameworks are printed in wax or plastics and then produced by casting using the lost-wax technique. In the direct method, the CAD data set is directly converted into a Co-Cr alloy object by laser sintering [30,31,32] (Figure 4). This method is currently still in the prototype stage. Recent publications have claimed advantages for laser sintering in digital manufacturing in terms of standardization, reduced production times, and easy transfer of digital data [33]. However, its economic viability is still critically assessed [34]. Additional research is required before this method can be definitely recommended. Particular attention must be paid to the retaining elements (clasps), as these are permanently exposed to high mechanical loads as they serve in their retaining and supporting function.

An in-vitro study by the authors, conducted at the Department for Dental Prosthetics University Hospital, Ludwig-Maximilians University Munich, examined the mechanical quality of cast versus laser-sintered clasps for cast-metal frameworks. The results of the study are very promising and show the high mechanical potential of laser-sintered clasps. The following key statements can be made on the basis of this study [35]:

- The required initial clasp withdrawal forces were attained by the cast and laser-sintered clasps alike. After artificial aging, the laser-sintered clasps exhibited no decrease in retention force.
- Pores and flaws were smaller and more evenly distributed overall in the laser-sintered clasps compared to the cast clasps.
- Laser-sintered clasps performed significantly better in the long term than cast clasps, with more than twice the latter’s survival rates. One reason could be the superior structural quality of the laser-sintered clasps.

#### 4.1.3. Hybrid Manufacturing 

**Figure 5:**
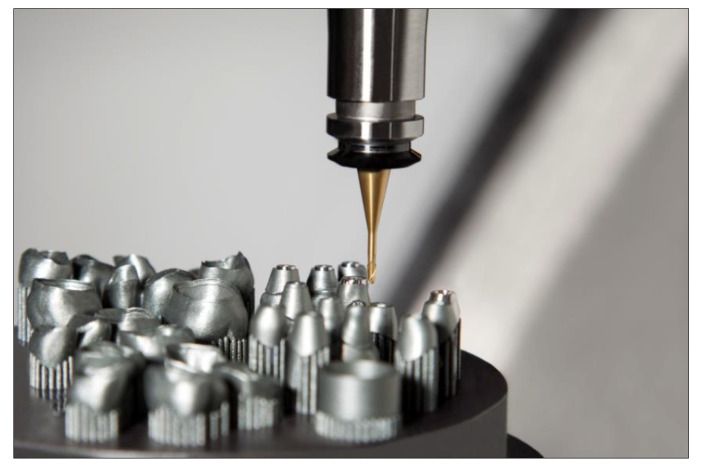
Hybrid manufacturing combines additive manufacturing. With CNC-milling (Source: Datron AG, Mühltal, Germany).

In digital dental technology, hybrid production is the term for a combination of additive and subtractive steps with a view to combining the efficiency of additive manufacturing with the precision of CNC milling [36,37]. Objects made using hybrid processes are characterized by improved surface structures, greater accuracy of fit, and lower cost (Figure 5). The company Datron (Mühltal, Germany) has been working on the implementation of dental manufacturing using hybrid technology for more than 8 years. A collaboration project by Datron, Concept Laser (Lichtenfels, Germany), and the Follow Me Technology Group (Munich, Germany) is working on mapping the hybrid workflow to standard milling machines through smart networking. An essential part of hybrid manufacturing is the transfer of the zero point (origo) from the additive process to the CNC milling unit. For this purpose, three measuring pins are built on the build platform during sintering. These pins are detected by the Datron D5 milling unit by means of an infrared touch probe developed especially for the hybrid manufacturing process, allowing the unit to determine the exact positions of the laser-sintered objects. The correction values are calculated directly by the unit, thus that no new CAM calculations are required. Since the objects remain firmly attached to the platform for post-processing (no pick-up via the grid structure), maximum positioning accuracy and precision are ensured. For implant superstructures, the screw hole is machined from the basal side via the screw access canal using special form cutters. Manufacturing costs can be reduced in the range of 30% to 50%, depending on the production volume.

### 4.2. Additive Manufacturing and Polymers

Several 3D printing technologies exist for the additive manufacturing of plastic objects [38,39,40] and exhibit, when compared to each other, different characteristics regarding speed, resolution, size, and process reliability depending on the underlying technology (Table 1). Currently, stereolithographic processes predominate in the dental sector, including classic stereolithography using a laser source (stereolithography, SLA) and the so-called mask exposure processes (digital light processing, DLP). In both processes, the object is solidified by the action of light in a vat of photopolymer.

For about 3 years now, 3D printers have been available that use low-cost liquid crystal displays (LCD). The technology is called direct ultraviolet printing (DUP); it uses the LCD displays for pixel-by-pixel exposure of the build platform. UV LEDs with a wavelength range of 395 to 405 nm are usually used for background lighting.

Direct 3D printing processes (material jetting, MJT) are also used in dental applications. A special process worth mentioning is multi-material 3D printing by Stratasys, which allows different colors and materials with different properties to be processed simultaneously in a single build. Material extrusion (MEX) processes such as fused-filament fabrication (FFF) or fused deposition modeling (FDM) are currently of lesser relevance on the dental market since they require long printing times and are restricted to lower resolutions. Of the technologies mentioned for the plastics sector, SLA, DLP, and MJT appear to be the most interesting from a technical and economic point of view [41,42,43,44,45,46].

#### 4.2.1. Stereolithography Using a Laser Source (SLA)

Stereolithographic systems, which use laser beams to solidify liquids, were the first 3D printing systems to appear on the market. Charles Hull had applied for a patent for the first stereolithography printer as early as in the 1980s. The first devices were very extensive—and expensive. The latest generation of stereolithographic printers, by contrast, has become quite economical. Formlabs (Sommerville, MA, USA) has been offering a 3D printer for dental applications for about five years now. This very affordable system is an ideal entry-level system for 3D printing technology, even if building takes much longer than with to DLP printers.

#### 4.2.2. Digital Light Processing (DLP)

Along with stereolithography, digital light processing is probably one of the most popular additive manufacturing processes in the dental sector right now. The design of a DLP printer is similar to that of an SLA printer, the main difference being the light source used. In the SLA printer, the photopolymer is cured with the help of a laser beam. DLP printers use projection technology from Texas Instruments instead, where short-wave light (currently used wavelengths: 380 nm and 405 nm) is guided through a digital micromirror device (DMD) that constitutes the core of the DLP technology. The system uses controlled square micromirrors with an edge length of approximately 16 µm. The light is guided optically either onto the build platform, which resides in a translucent vat of photopolymer (photopolymer bath) or onto a diffuse surface (absorber). This is made possible by tilting the individual micromirrors in the unit, which are triggered by forces exerted by electrostatic fields [47,48]. The exposure mask is projected onto the build platform through an optical lens, causing the photopolymer to cure at the exposed areas. After each exposed mask, the build platform moves along the *z*-axis, and new material flows into the space beneath the object and can be exposed with the next mask. When using DLP technology, the building time is, therefore, almost independent of the objects produced, the decisive factor being the dimension of the object along the *z*-axis.

##### Resolution of DLP Printers

One micromirror corresponds to one image point (pixel). Since a DMD has a limited number of these micromirrors, when the build platform is increased in size, edge lengths along the x and y axes also increase, resulting in lower precision. There are currently three ways to, nevertheless, realize larger build platforms, although the first one will not find its way into standard lab printers for the time being on account of the cost involved:

- Using a DMD chip with higher resolution (e.g., 4K resolution)

Less expensive DLP printers use DMD chips with lower resolution (e.g., 1280 × 720 pixels) and a correspondingly smaller footprint. If DMD chips with high resolution (e.g., HD 1920 × 1080 pixels) are used, greater object accuracy can be achieved with the same footprint. When using 4K DMD chips (3840 × 2160 pixels), it is possible to achieve high resolutions while maintaining a large build area (e.g., Rapid Shape D70+; Rapid Shape, Heimsheim, Germany) [49]. However, the prices for 4K DMD chips are still very high.

- Two DLP projectors with HD resolution connected in parallel

This approach creates a “joint” on the build platform caused by the use of two light sources. As a result, no objects can be printed that are positioned across the projection field. Example: Rapid Shape D40 II (Rapid Shape) [50]

- Moving DLP projectors (W2P Engineering, Vienna, Austria)

DLP projectors whose optical subsystem moves below the material vat are able to expose a larger area [51]. One advantage of the Moving DLP is that the object will feature no joint line and that, consequently, the entire extent of the build platform can be used at full resolution. This makes for a higher resolution, greater printing accuracy, and better utilization of the capacity of the device.

- Prodways MovingLight technology (Prodways Group, Paris, France)

The MovingLight technology was developed and patented by the French company Prodways [52]. This AM technology is based on the DLP process. It differs from its competitors’ approaches in that the projector is not rigidly fixed in one location within the printer but moves around across the complete working area in several steps, achieving high resolutions (42 µm) and high accuracy despite the extensive build platform [53]. Examples include Prodways’ ProMaker LD10 Dental Plus, LD10 Dental Models, LD20 Dental Plus, and LD20 Dental Models. The latter two have two movable projector heads, reducing build times by another 40%. For example, it takes about 1 hour to print 55 dental arches.

##### DLP Printer Build Process Optimization

The DLP printers also use various techniques to detach objects from the material vat during the build process. This detachment occurs after each exposure cycle when the build platform is lifted along the *z*-axis. Four different techniques are applied:

- Fixed intervals

The build platform covers a defined path in a defined time after the exposure cycle. The path/time ratio here remains the same within a build process, even if the object could be removed sooner in the process (e.g., when using fewer support structures). The fixed-interval principle is very simple, but the duration of the building processes is not altered.

- Force Feedback technology (Rapid Shape, Heimsheim, Germany)

The force needed for detachment can be measured via force sensors. The smart control technology is then used to calculate an optimum path/time ratio, which speeds up the building process [54]. A particular advantage is that the separation process is controlled and gentle. The patented Force Feedback technology is used, for example, by the Rapid Shape D30.

- Vat deflection feedback system (VDFS; W2P, Vienna, Austria)

The patented vat deflection feedback system uses an additional sensor to speed up the building process. In addition, the material tray can be deformed (FlexVat), allowing the detachment force to be minimized and resulting in increased printing speed and quality [55,56].

- Continuous direct light processing (CDLP; Carbon3D, Redwood City, CA, USA)

In 2015, Carbon3D first released information on its patented continuous liquid interface production (CLIP) technology, which is classified as a CDLP process [57,58]. Unlike the incremental build-up of objects in DLP printers, the CLIP process involves a continuous build process without the steps normally required to detach objects from the build platform in DLP printing. This process is enabled by the fact that there is an oxygen-rich zone (“dead zone”) immediately above the build platform where no curing of the photopolymer takes place. Oxygen is conducted into the “dead zone” through a window that is permeable to oxygen. Since there is no adhesion of the object to the build platform, a continuous build process is possible. The result is extremely high build speeds with high object precision and continuous object geometries along the *z*-axis. Examples of dental applications include additively manufactured Lucitone Digital Print denture bases from DentsplySirona (York, PA, USA) or bite splints made of KeyPrint or KeySplint Soft Clear, both additively manufactured using a Carbon3D printer.

#### 4.2.3. Material Jetting (MJT)

In material jetting, the material is applied directly to the build platform via the print head (similar to the 2D printing process) and then cured in an intermediate exposure step, building up the object layer by layer. The best-known representative of this technology is the Polyjet method (Stratasys, Eden Prairie, MN, USA), characterized by an extremely fast build process and high precision [41,42,43]. A special feature is multi-material 3D printing, where five different grades of materials can be printed in more than 500,000 colors [59,60,61]. The Stratasys product portfolio includes, for example, the J720 Dental or J750 Digital Anatomy printers that operate in multi-material multicolor mode.

#### 4.2.4. Useful Indications for AM of Polymers

- Model fabrication based on intraoral scan data

**Figure 6:**
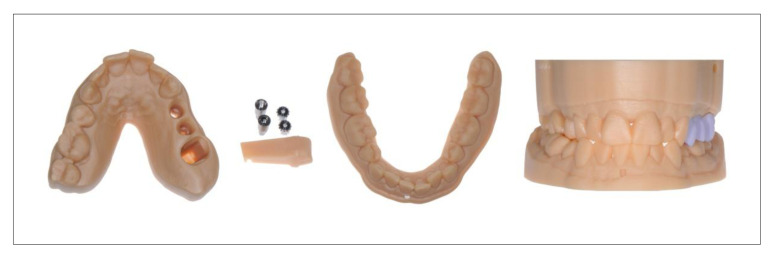
Additive manufactured models for implantology.

Due to the high efficiency of DLP printers in combination with high precision, the digital fabrication of master models and segmented models is one of the primary domains of DLP printers [62]. In particular, the additive manufacturing of models for oral implantology would appear to be an interesting application for these systems (Figure 6). Precise positioning of the laboratory analogs in the printed model is crucial, as it has a decisive influence on the proximal and occlusal fit of the restorations.

- Templates (drilling stents) for guided implant surgery

**Figure 7:**
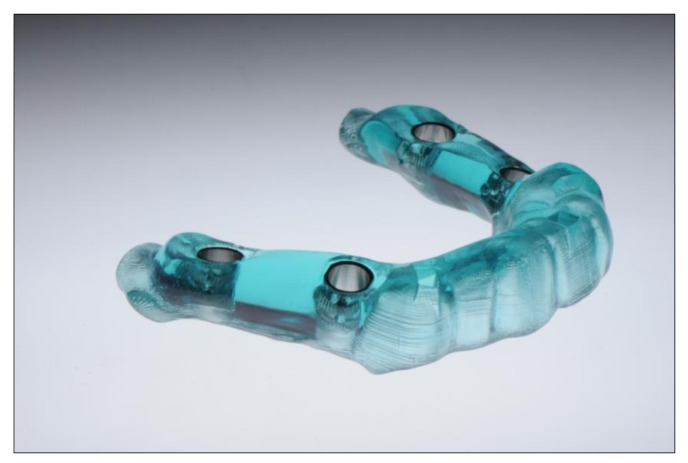
3D printed surgical guide with drilling sleeves.

Software developments in recent years have made it possible to overlay (match) volume data sets from radiology (DICOM) with surface data sets (STL) from the laboratory or from intraoral scanners. This allows optimizing the implant position, taking into account anatomical, surgical, and prosthetic aspects. The planned positions are then realized with the help of a surgical template inserted into the patient’s mouth. DLP printing technology offers particular advantages here, as it allows very quick production at low costs (Figure 7). Unlike subtractive methods, there are no restrictions on the design of the three-dimensional geometry [63].

- Custom impression trays

**Figure 8:**
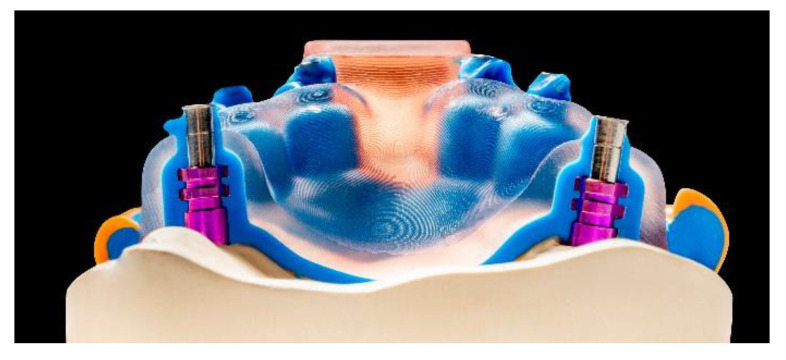
Cross-section through an implant impression tray on the implant model (Source: Shera Werkstofftechnologie, Lemförde, Germany).

The production of custom impression trays is made particularly enticing by DLP printing technology due to the sped of this technology. The CAD software solutions available on the market allow custom impression trays to be designed with optimum fit parameters in just a few steps, saving considerable time, especially when undercuts are blocked out virtually and can be dimensioned more precisely. It is important to avoid irreversible deformation of the impression during removal [64]. Despite their technical advantages, it should be pointed out that the materials currently intended for the fabrication of functional impression trays are expensive, making them viable only for use in implant impression trays (Figure 8). It would appear advisable to combine them with digital implant planning, as digital models will already be available in this context, and the position of the planned implants can be used as a basis for tray fabrication.

- Production of occlusal splints

**Figure 9:**
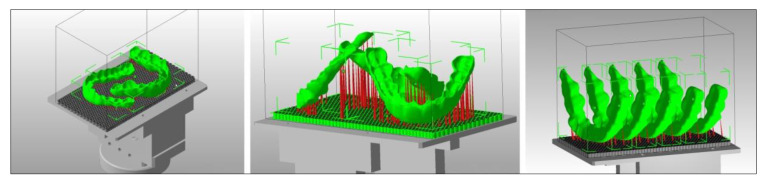
Different orientations for occlusal splints on the build platform.

In addition to production using the scatter-and-press method or subtractive milling, it is also possible to produce precision-fit occlusal splints by 3D printing. However, in addition to the overall production accuracy, the quality of the material and the associated long-term stability and biocompatibility are determining factors. No long-term clinical experience with additively manufactured occlusal splints has as yet been reported. At the same time, it is necessary to investigate the elution behavior of additively manufactured occlusal splints under laboratory and oral conditions [65]. Comparisons with current procedures would be desirable to decide which manufacturing process yields the best long-term results. The bar has generally been set very high for homogeneity and biocompatibility as achieved by high-performance polymers machined in the subtractive CAD/CAM process (e.g., milled splints). Factors such as the positioning and alignment of the objects and their influence on accuracy, stability, and durability must also be investigated. The working angle on the build platform and, hence, the direction of the layers seem to be of particular importance here (Figure 9). Initial studies have shown that 3D-printed occlusal splints are similarly accurate as CAD/CAM-milled splints but exhibit higher material wear and less favorable material properties [66,67,68].

- Production of realistic training models

**Figure 10:**
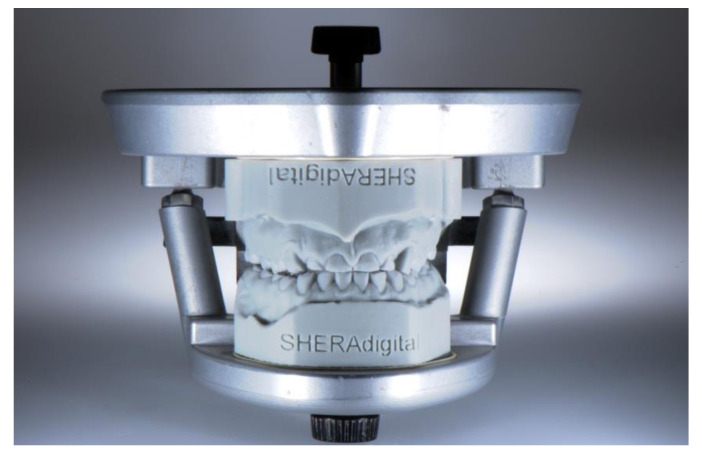
3D printed realistic training models in a standard phantom head.

Realistic patient models for training and continuing education courses have been developed by the Department for Dental Prosthetics of the University of Munich. These models can be fixed on standard phantom heads (Figure 10). Their design is based on scanned models, with connection geometries (threading, anti-rotational features) added in the CAD software. In order to save on weight and material, the models are hollow on the inside and possess a reinforcing grid. After adding the support structures and subsequent slicing, the models were printed on the SheraPrint D30. The SheraPrint-model was used as the material for the models, a material that is also excellently suited for the preparation of various restorative shapes with irrigated rotary instruments. These models can be used to easily simulate the cementation of various restorations in the phantom head [61]. 

**Figure 11:**
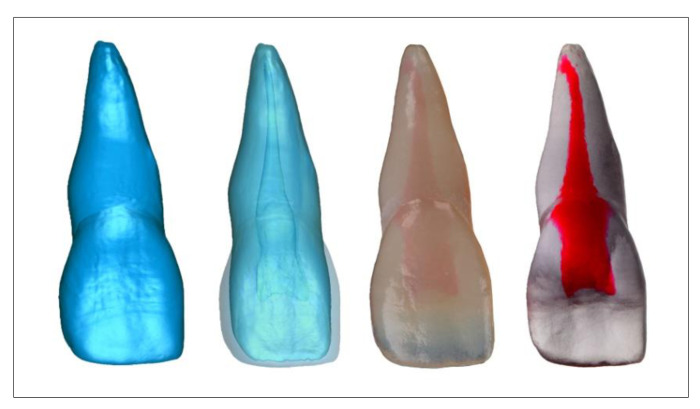
Nature-identical simulation teeth.

**Figure 12:**
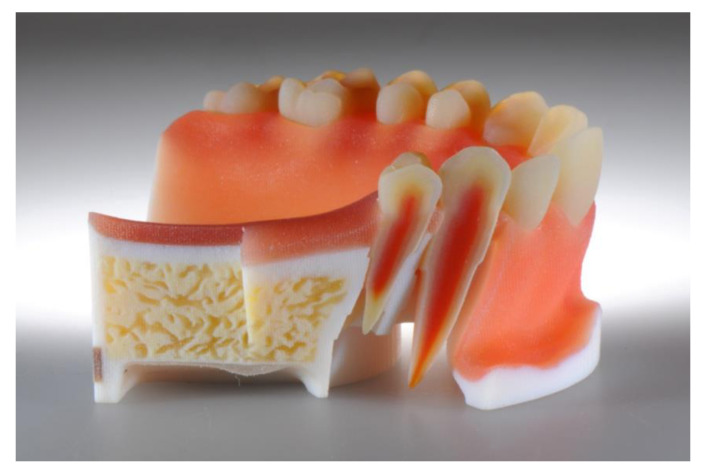
Multi-layered model of the complete jaw for surgical simulations and trainings.

The next stage of development for the production of training models are multi-layer models. This multi-layering can refer to tooth structures as well as to layered structures of the entire jaw. Such models are extremely versatile to use; they may cover nature-identical simulation teeth for endodontic exercises [69] (Figure 11) to multi-layered models of the complete jaw for surgical simulations and trainings [70] (Figure 12).

- Production of graphic 3D models (3D Medical Print, Lenzing, Austria)

**Figure 13:**
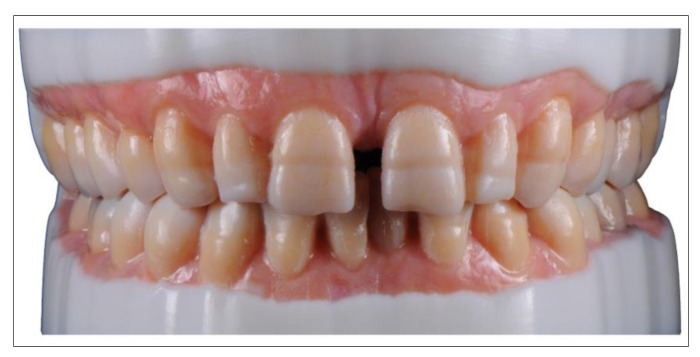
3D printed graphic 3D model based on 3D data from an intraoral 3D scanners.

Several intraoral 3D scanners now allow the digital capture of shade information in addition to surface data. Available file formats include PLY, OBJ, and VRML. Using Polyjet technology, it is possible to convert these data into physical models. The pertinent shade information is geometry-related, i.e., the two-dimensional shade information is uniquely assigned to 3D surfaces. Model builder software is used to generate a virtual shade model, which is then converted into a physical shade model using multi-material 3D printing (Polyjet technology; Stratasys, Rheinmünster, Germany) (Figure 13). Since the transfer of shade information is not possible with analog impressions, graphic 3D models are a veritable “killer application.” Data generation and model production mandate the use of a digital workflow. In the future, new possibilities will emerge here that will be associated with enormous improvements and simplified procedures, especially for highly esthetic dental restorations [2,71]. 

**Figure 14:**
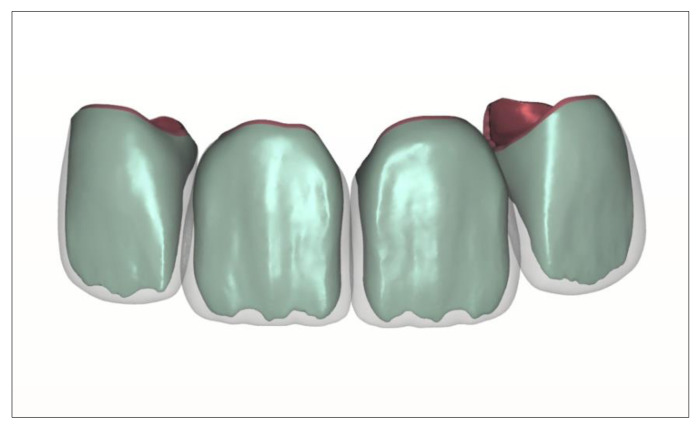
CAD–construction of 4 upper incisal crowns using the tooth-structure database.

Another possible application of multi-material 3D printing in the dental field could be the fabrication of multi-layered dentures made from different materials. With regard to the identical reproduction of natural teeth by crowns or bridges, this technology is currently in the prototype phase. It is based on the tooth-structure database, according to Schweiger [72,73,74], which allows the multilayer structure of natural teeth to be copied and the data thus generated to be used in an additive manufacturing process (Figure 14). The ultimate aim is to produce biomimetic dental restorations that reflect the multi-layered three-dimensionality as well as the complex mechanical and optical properties of natural teeth. Taking into account the light-optical properties of the different tooth layers (pulp, dentin, enamel), an identical esthetic reproduction of natural teeth can be achieved.

**Figure 15:**
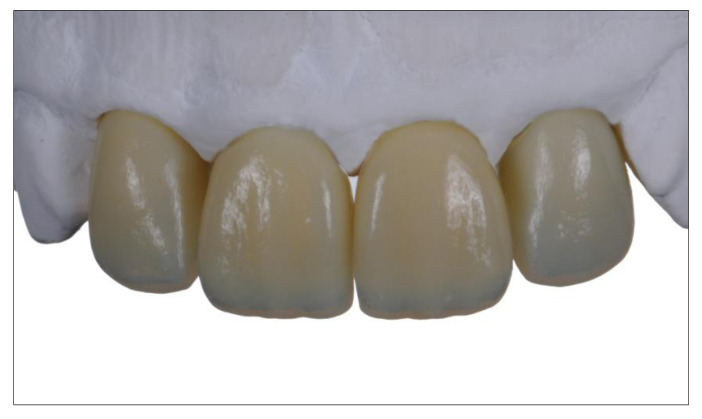
3D printed multi-layered upper incisal crowns.

Current research at the Department for Dental Prosthetics at the University of Munich uses data from the tooth-structure database in a Stratasys Polyjet process to implement the concept. At present, the process allows the fabrication of esthetic try-in crowns or bridges from light-polymerizing resins (Figure 15). The materials used are approved for use in the mouth for up to 24 h, permitting the evaluation of functional and not least esthetic criteria. 

Since the layering process includes no analogous steps, the result is not influenced by manual imponderables. The composition of the printing materials in combination with the three-dimensional multi-layer structure of the denture are the only determinants of the structural and esthetic results. Fine-tuning the material composition in the multi-material 3D printing process is likely to permit fine-tuning of optical properties in the future. For example, various mixtures for the enamel compound are currently being tested in in-vitro studies to replicate the light transmission behavior of natural tooth enamel as closely as possible. Likewise, the shade and translucency of different dentin qualities can be adjusted by mixing. The layered 3D design of the restorations is reproducible thanks to the digital design process. After the try-in, the layered structure can be transferred to the final ceramic restoration, for example, by way of subtractive manufacturing [61].

- VarseoSmile Crown plus–3D printing of permanent single-tooth restorations (Bego, Bremen, Germany)

**Figure 16:**
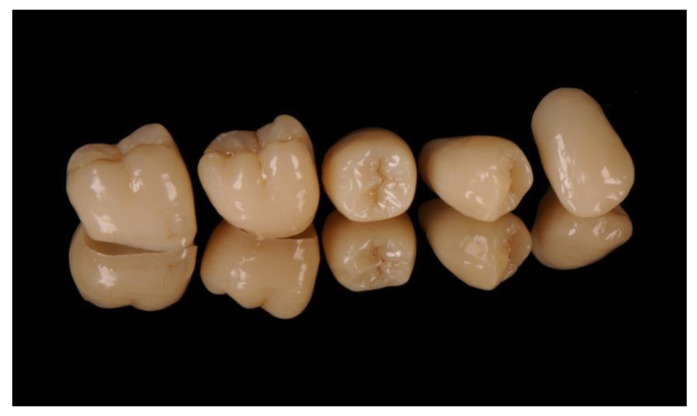
Single-tooth crowns printed with VarseoSmile Crown ^plus^ (Bego).

The question of whether definitive dental restorations can be produced by a 3D printer can be answered by looking at the findings of materials science regarding 3D printing materials. The requirements of materials for dental prostheses permanently installed in the mouth are by necessity high. Definitive restorations require the use of materials that can withstand both high mechanical stress and the various chemical processes present in the oral cavity. No harmful substances must be released during the wearing period, and the materials must have a smooth surface to forestall bacterial deposits (plaque). In addition, a practical and economical manufacturing process must be available that can ensure precision in the micrometer range. Since February 2020, Bego has been offering the world’s first method for manufacturing single-tooth restorations using 3D printing and a ceramically reinforced hybrid material. The Bego VarseoSmile Crown ^plus^ can be used to produce single-tooth crowns, inlays, onlays, and veneers using an additive process. The material has been extensively studied in scientific testing and has yielded excellent results. In particular, its fracture load (at baseline and after artificial aging), abrasion resistance, long-term stability of the cementing agent, solubility, and cytotoxicity were investigated [75]. Production in the Bego Varseo XS, which is a low-cost, high-resolution DLP 3D printer with excellent detail resolution, appears particularly interesting. Up to 20 individual restorations can be printed simultaneously on the build platform. The printer is network-compatible, facilitating fast and uncomplicated data exchange with CAD PCs. After the printing process, cleaning is carried out using ethanol and air-abrading using gloss beads (e.g., Perlablast micro; Bego). The restorations are then post-polymerized in the Bego Otoflash light-curing unit. As the surface of the printed restorations is smooth and homogeneous, the finishing step can be limited to smoothing the surface and subsequent polishing. Alternatively, the polymerized restorations can be customized using commercially available composite-resin stains (Figure 16). Bego VarseoSmile Crown plus restorations are cemented with self-adhesive luting materials (e.g., RelyX Unicem; 3M, Seefeld, Germany) or with luting composites with a separate primer (e.g., Variolink Esthetic DC and Monobond Plus; Ivoclar Vivadent, Schaan, Liechtenstein). The VarseoSmile Crown plus hybrid material is available in 7 shades (A1, A2, A3, B1, B3, C2, D3).

### 4.3. Additive Manufacturing and Ceramics

A number of different build-up methods now allow even ceramic materials to be processed using either indirect or direct techniques.

**Indirect technique**

- Trix print process by Dekema (Freilassing, Germany)
- IPS e.max Digital Press Design–Wax Tree by Ivoclar Vivadent (Schaan, Liechtenstein)

**Direct technique**

- SLA process, e.g., 3DCeram (Limoges, France)
- DLP process, e.g., LCM (lithography-based ceramic manufacturing, LCM) by Lithoz (Vienna, Austria)
- Material extrusion (fused-filament fabrication, FFF; paste-extrusion modeling, PEM)
- Material jetting/nanoparticle jetting, e.g., XJET (Rehovot, Israel)
- Binder jetting, e.g., 3D Systems (Rock Hill, SC, USA)
- SLS process (research project at the Department for Dental Prosthetics of the University of Munich, the Friedrich Baur Institute for Biomaterials at Bayreuth, Germany, and Concept Laser at Lichtenfels, Germany)
- LOM process (laminated object layering)

#### 4.3.1. Indirect 3D Printing of Ceramics Example: Dekema Trix Print

Dekema (Freilassing, Germany) uses a novel approach to pressable ceramics with its innovative Trix system. It combines the advantages of digital design with the unbeatable efficiency of proven ceramic pressing technology. The system maps the entire pressing workflow digitally, from wax-up to the pressing itself. The individual steps are explained below using the example of several partial crown restorations.

**Scanning and CAD design**

The oral situation can be digitally acquired directly, by means of an intraoral scanner, or indirectly, by scanning a master cast after taking an analog impression. The digital pressing technology is suitable for both acquisition methods. The partial crowns can be efficiently using standard CAD software tools.

**Automatic addition of sprues and placeholders for up to three pressing plungers**

After selecting the objects to be pressed from the respective CAD system, Trix CAD automatically designs the complete wax-up, including the placeholders for up to three pressing plungers, in order to press up to three pressing pellets (which can be of different shades) in one process. Trix CAM determines the required layer pattern and sends it to the Dekema Trix print 3D printer.

**3D printing using the Dekema Trix print 3D printer**

The sliced layer data are printed on the build platform of the Trixpress muffle system. The associated Trix cast printable burnout material is also made by Dekema.

**Investing and pressing**

**Figure 17:**
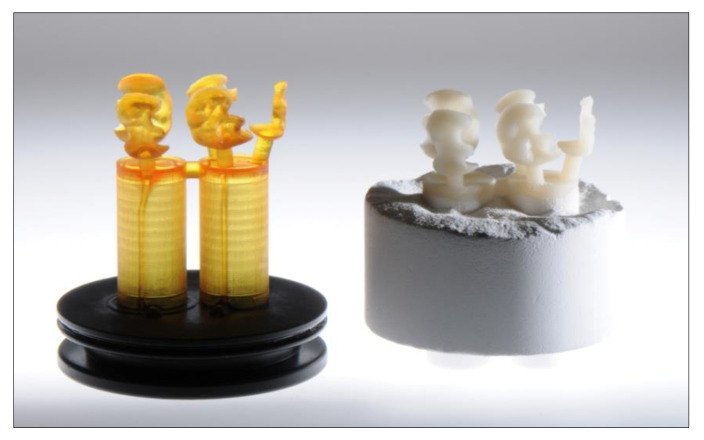
Comparison of 3D printed and pressed ceramic inlays.

3D printing is followed by cleaning and curing the objects and investing them in the Trixpress muffle. After heating in the preheating furnace and residue-free calcination, the pressing ceramic is inserted into the muffle and usually pressed with the Trixpress punches (Figure 17). The project-specific pressing program has already been streamed from the Trix CAM to the Austromat 654i for this purpose. Alternatively, the data can also be transferred by way of a USB stick.

**Finishing and glazing**

After pressing, the partial crowns are finalized following standard procedure; there is no difference between this workflow and the analog workflow. When working with a completely digital workflow, it is recommended to record the scan data of the dentition by means of a 3D-printed model thus that it is possible to check the fit along with the proximal and occlusal contacts. Staining and glaze firing then completes the fabrication of the partial crowns.

#### 4.3.2. Direct 3D Printing of Ceramics Example: LCM Technology

No market-ready applications are yet available for direct 3D printing in the dental realm. The most advanced approach is probably the patented LCM process by Lithoz (Vienna, Austria) [76,77]. We will illustrate the current state of the art in dental zirconia 3D printing using the example of a mandibular molar crown. After scanning the jaws and CAD-designing the restoration, the fully contoured crown is fabricated using Lithoz’ lithography-based ceramic manufacturing (LCM) technology. The LCM process is based on digital light processing (DLP). Here a photosensitive ceramic slurry is selectively cured, achieving a high filler content and a dense packing of the ceramic particles in the pre-sintered blank. This is necessary to produce defect-free and dense ceramic objects. The polymer network connects the ceramic particles. For dental applications, Lithoz has developed the CeraFab 7500 Dental 3D printer. The fully contoured posterior crown was made from LithaCon 3Y 230 (zirconia stabilized with 3 mol-% yttria-stabilized zirconia, 3Y-TZP). The printing process took approximately 7 h for 20 crowns, for a printing time of 21 min per crown.

After the additive manufacturing process, the crowns are available as “green bodies” that still contain the organic binder material, which must be removed in the next step—thermal debinding at 1000 °C over a period of several hours. This creates the so-called “white body,” which no longer contains any binder and will already have formed solid sintering bridges that prevent the object from disintegrating. At this point, individual staining is performed using staining solutions, with three variants being available:

- Immersing the crown in the staining solution
- Custom painting of the crown using a brush and staining solution
- A combination of the two

**Figure 18:**
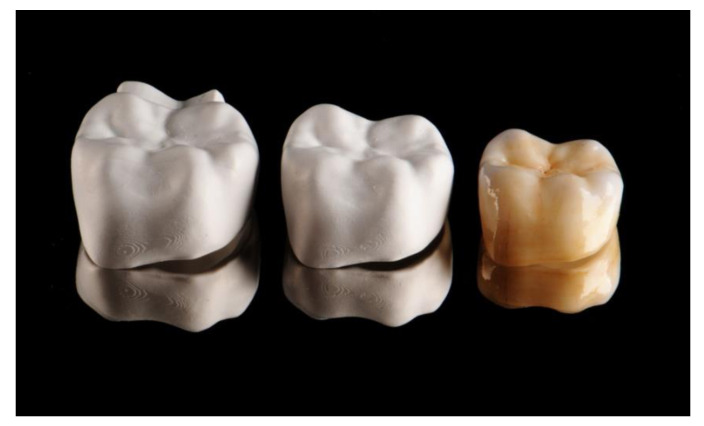
3D printed Zirconia crown in the green and the white state and finally sintered.

The combination variant has shown itself to be the preferred variant. Here, a basic stain is achieved by immersion, followed by individual characterization using various intensive staining solutions, particularly at the crown margin and in the incisal/occlusal area. After staining, it is important that the crowns are dried before the final sintering step, ideally using infrared light. The sintering process is carried out at 1600 °C, at a heating rate of 8 °C/min and a holding time at the final temperature of 2 h. The cooling rate was also 8 °C/min down to 500 °C with subsequent ambient cooling to room temperature. The crowns are finalized with a stain firing and a glaze firing at 770 °C; IPS e.max Ceram Stains were used for this purpose in the case illustrated (Figure 18).

**Assessment of the final result**

The crown was made from 3Y-TZP using Lithoz’ LCM process. This classic zirconia was originally designed intended for the fabrication of crown or bridge frameworks, which were manually veneered using a ceramic material made of silicate ceramics. The translucency of the frameworks was, therefore, low. Nevertheless, the LCM process can achieve pleasing esthetics even with fully contoured crowns. The excellent reproduction of the sharp-edged crown margins and the exact reproduction of the occlusal surfaces with a well-defined and natural representation of the fissures were particularly striking. Since subtractive machining invariably requires crown margins to be reinforced and the occlusal fissures will always be rounded due to the finite diameter of the burs, additive manufacturing proves advantageous here.

#### 4.3.3. Multi-Material 3D Printing of Ceramics

The most interesting development in the field of additive manufacturing using ceramics is multi-material 3D printing. The first prototypes were presented by the WZR company in 2014 [15], which combined two processes, namely binder jetting (BJT) and material jetting (MJT). Here, particle-filled inks are applied directly to the powder bed via the print head. If, for example, a different material is selected for the ink it is possible to alter the structural composition of the workpiece. Inks filled with metal particles can also be injected into a ceramic powder bed, thus that, for example, an object made of silicate ceramics can be built that integrates electrical conductor paths in silver.

The latest development in this field was presented by Lithoz in mid-2020. A specially developed LCM printer (CeraFab Multi 2M30) makes it possible to produce objects from different materials in a single printing process. It is possible not only to combine different ceramics but also to create ceramo-metal and ceramo-polymer objects. Material combinations currently include four variants [77]:

- Two materials in a single layer
- A denser material combined with a second porous material
- Two-phase or multi-phase materials with gradual variations in composition
- Gradual variations in both density and composition

These currently available options presage the enormous potential of this technology. It is likely that this will also affect additive manufacturing for dental applications.

## 5. Limitations of 3D-Printing

Basically, you can distinguish between 3D-printers for hobby use and for professional use. Practical application has shown that the relatively inexpensive printers for hobby users often show poor printing results, especially noticeable gradations in the FFF printers due to the filament fibers. For this reason, the devices available for dental use are mostly expensive but show good final results. However, even the printers for professional use generate a more or less pronounced gradation in the Z-direction. This is largely dependent on the thickness of the individual layers. The thinner the building layer, the lower the graduation, but also the longer the processing time. A further limitation is the maximum achievable build speed and the size of the build space. Newly developed technologies in the field of component detachment (see Section 4.2.2) in particular can solve the speed problem and lead to extremely high construction speeds. There are also limitations in the materials that can be used for 3D-printing. Especially in the field of polymers, printers based on photopolymers are predominantly used in dental technology, especially in the field of VAT polymerization (SLA, DLP, DUP). This greatly reduces the range of resins that can be used, resulting in significant disadvantages here compared to standard manufacturing processes (e.g., CNC technologies, analog manufacturing techniques). A possible solution to this problem could be the so-called "drop-on-demand" technology in which thermoplastics approved for medical technology are melted from a granulate and applied dropwise in a plastic state to the build platform. The achievable surface quality of this technique differs substantially from the results from filament printers. Further on, there is only little data regarding the behavior of 3D printed devices or restaurations in the oral cavity. Data on plaque formation, elution behavior, and general biocompatibility of 3D printed polymer materials are scarce [65,78], and further data on specific materials are urgently required. 

## 6. Outlook

Additive processes have the significant advantage that an object´s properties can be individually influenced during the construction process. This applies to mechanical and esthetic properties alike. In subtractive processes, by contrast, these characteristics are predetermined by the manufactured milling blank. 3D-printing thus gives users an enormous range of choices as early as during the design process. On the other hand, the precision and efficiency of subtractive machining are extremely high, thus that the combination of both manufacturing techniques seems to make eminent sense. 

In addition to the production of auxiliary systems (surgical guides, models, individual impression trays) and fixed dentures, there is a trend towards 3D-printing in the field of removable dentures. RPD’s made of CoCr using additive technologies have already found their way into dental laboratories and practices. Currently, more and more publications on additive manufacturing of complete dentures are being published [79,80,81,82]. The results regarding mechanical strength, fit, and surface quality are promising. Since the denture bases have large area contact with the oral mucosa, biocompatibility must be critically examined. In particular, elution behavior and cytotoxicity must be investigated before a final assessment is made [65,78].

Finally, there are areas in which the classic analog processes are unbeatable in terms of economy, for example, ceramic pressing. However, here, too, integrating digital steps can be useful. With further advances in the additive manufacturing of ceramic restorations, innovative approaches to the production of natural-looking dental restorations will soon arise. Digital acquisition of three-dimensional tooth layering using NIRI technology—a likely future achievement—could be a foundation of this technique, together with tooth-structure databases [72,73,74]. Additive technologies such as the Lithoz LCM process are the ideal manufacturing routes to achieving this goal. Gradient technologies can be individually adapted to restoration geometries and offer unimagined design freedom in three-dimensional space, impossible to achieve with conventional technologies—all within the scope of patient-focused, individualized, and personalized dentistry.

## Figures and Tables

**Table 1:** Characteristics of 3D printing technologies for plastics used in the dental sector.

	Filament-Based 3D Printing	Light-Based 3D Printing			Material Jetting
	FDM/FFF	SLA	DUP	DLP	MJT
Speed	medium	medium	medium	high	high
Resolution	low	high	medium	high	high
Size	scalable	scalable	scalable	scalable	scalable
Process reliability	medium	medium	low	high	high
Cost	low	medium	low	medium to high	high
